# X-ray phase-contrast 3D virtual histology characterises complex tissue architecture in colorectal cancer

**DOI:** 10.3389/fgstr.2023.1283052

**Published:** 2023-10-24

**Authors:** Angelika Svetlove, Titus Griebel, Jonas Albers, Lorenzo D’Amico, Philipp Nolte, Giuliana Tromba, Hanibal Bohnenberger, Frauke Alves, Christian Dullin

**Affiliations:** ^1^ Translational Molecular Imaging, Max Planck Institute for Multidisciplinary Sciences, Göttingen, Germany; ^2^ Cluster of Excellence Multiscale Bioimaging: From Molecular Machines to Networks of Excitable Cells, University Medical Center Göttingen, Göttingen, Germany; ^3^ European Molecular Biology Laboratory Hamburg, Hamburg, Germany; ^4^ Elettra Sincrotrone Trieste S.C.p.A, Trieste, Italy; ^5^ Department of Physics, University of Trieste, Trieste, Italy; ^6^ Faculty of Engineering and Health, University of Applied Sciences and Arts, Göttingen, Germany; ^7^ Institute for Diagnostic and Interventional Radiology, University Medical Center Goettingen, Goettingen, Germany; ^8^ Institute of Pathology, University Medical Center Göttingen, Göttingen, Germany; ^9^ Clinic for Haematology and Medical Oncology, University Medical Center Göttingen, Göttingen, Germany; ^10^ Translational Lung Research Center, Heidelberg University Hospital, Heidelberg, Germany; ^11^ Clinic for Diagnostic and Interventional Radiology, University Hospital Heidelberg, Heidelberg, Germany

**Keywords:** microCT, virtual histology, synchrotron, colorectal cancer, digital pathology, phase contrast

## Abstract

Precise morphological analysis of tumour tissue samples is crucial for accurate diagnosis and staging of colorectal cancer (CRC), but remains limited by the 2D nature of conventional histology. Our aim is to offer a 3D representation of tissue samples by means of X-ray-based imaging to facilitate the evaluation of clinically relevant features in cancer tissue, a process that is currently subject to various restrictions. In this study, we show that propagation-based synchrotron radiation-based free propagation phase-contrast microcomputed tomography (SRµCT) is suitable for the generation of 3D tumour volumes with 2-µm voxel size using standard formalin-fixed, paraffin-embedded tissue from CRC patients and provides sufficient contrast for virtual histology. We demonstrate that, using an existing registration pipeline, a 2D histologic haematoxylin–eosin slice can be placed in the context of the 3D µCT volume. The precisely registered histologic section can then be used as a “seed point” for the segmentation and depiction of major histologic features. This approach allows for a more comprehensive understanding of the organisation of the tumour in space with respect to other structures such as vessels, fat, and lymph nodes, and has the potential to improve patients’ prognostic outcomes.

## 1 Introduction

Colorectal cancer (CRC) represents the most common malignancy of the gastrointestinal tract, exhibiting the third-highest incidence rate amongst men (accounting for 746,000 cases worldwide and 10% of all cancer diagnoses) and the second-highest incidence rate amongst women (representing 614,000 cases and 9.2% of all cancer diagnoses in 2012) (1). CRC led to approximately 0.92 million deaths in 2020 (2), and it is estimated that by 2030 the number of deaths due to CRC will have risen to 1.1 million per year (3).

Precise local staging, along with assessment of metastasis and lymph node involvement in CRC, is crucial for prognostic estimation and for well-founded treatment decisions (which are considerably dependent on the diagnosed stage) (4–8). The need for improvement is emphasised by the significant understaging currently observed in both the T and N stages, which can, for example, lead to under-identification of patients who would benefit from adjuvant treatment (9). However, treatment response and the prognostic survival quality of CRC patients with the same pathological stage may differ due to tumour heterogeneity.

Currently, prognostic features include histologic type, invasion pattern, differentiation grade, lymphovascular and venous invasion, perineural invasion, tumour deposits, and tumour budding (10). Grading, as a stage-independent prognostic factor, reflects gland formation in adenocarcinoma, the most frequent histologic type of CRC, and comprises three categories, namely grade I, grade II, and grade III: well-differentiated adenocarcinoma, moderately differentiated adenocarcinoma, and poorly differentiated adenocarcinoma, respectively (11). The diagnosis of vascular invasion, also a substantial determinant of therapy and prognosis, remains challenging with the use of conventional haematoxylin–eosin (HE) staining alone. Usually, other types of staining or immunohistochemistry are required for an accurate evaluation (12), which could be facilitated by a 3D representation of the tissue sample. Molecular characteristics represent another aspect of CRC classification, especially microsatellite instability (MSI) and KRAS/BRAF mutational status, which are also both substantial determinants of treatment and prognosis (13, 14).

Although some of the features assessed in pathological examination, such as gland formation and tumour grade, can be viewed clearly using traditional immuno-/histochemistry, conventional histologic assessment remains inherently 2D, even when multitudes of sections are reviewed. Other clinically important features such as tumour budding, the invasion front, and vascular infiltration cannot be assessed with ease. Although an experienced pathologist may extrapolate based on multiple 2D histologic slices, a true 3D representation is difficult to accomplish.

This challenge can be addressed through the use of X-ray-based microcomputed tomography (µCT), a non-destructive isotropic 3D imaging technique, which is becoming increasingly popular for the analysis of soft-tissue samples in (pre-)clinical research (15–18). µCT imaging achieves high resolution by taking advantage of optimised X-ray sources with narrower focal points as well as smaller pixel detectors. Although commercial lab-based systems can acquire datasets at high resolutions, they require additional staining of the soft-tissue specimens using compounds such as iodine or phosphotungstic acid in order to reach sufficient tissue contrast levels (19). In addition, due to the low photon flux of the micro/nanofocus X-ray tubes utilised in lab µCT systems, the scan duration can be multiple hours per sample (20, 21).

Novel X-ray imaging techniques exploiting effects such as scattering and phase shift rather than attenuation, which defines contrast in classical CT imaging, provide increased soft-tissue contrast and enable the label-free assessment of such specimens in 3D. Among these techniques, propagation-based imaging (PBI) is the simplest. PBI requires at least partially coherent X-ray sources and a sufficient sample-to-detector distance. Although PBI is technically feasible with laboratory sources (22, 23), it is widely used in combination with synchrotron light sources. Synchrotron radiation-based free propagation phase-contrast µCT (SRµCT) is known to be compatible with the standard histologic protocol. Formalin-fixed and paraffin-embedded (FFPE) tissue samples can be imaged directly in the paraffin block prior to sectioning and staining (19). In addition, SRµCT imaging can be performed much more quickly than imaging using lab systems, due to the extremely high photon flux in the synchrotron setting (24).

In this report, we demonstrate that features such as tissue layers and lymphatic/blood vessels can be effectively visualised and evaluated in 3D via SRµCT of the entire paraffin block of CRC tissue samples obtained during surgical resection. By fusion of histologic slices with the context of the SRµCT dataset, we show that histology-specific labels can be assigned to the µCT structures and continuously traced throughout the volume.

## 2 Materials and methods

### 2.1 FFPE CRC tissue samples

Patient CRC tissue specimens (maximum size 3 mm× 25 mm× 25 mm) were taken during surgical procedures, formalin fixed, and embedded into paraffin blocks in accordance with the standard clinical pathology workflow. The tissue blocks were provided by the Institute of Pathology, University Medical Center Göttingen, Germany. The blocks were archived and stored at room temperature, with storage times ranging from 5 years to 12 years. All patients gave written informed consent for the use of their pathology specimens for research purposes. A summary of the tissue origins, initial diagnosis, and the UICC classification can be found in **Table 1**. The study was conducted with the permission of the corresponding hospital ethics committee (Ethik-Kommission der Universitätsmedizin Göttingen, permission number 24/4/20) and in accordance with the Declaration of Helsinki.

**Table 1:** List of the scanned CRC samples.

Patient no.	Histology	Localisation	UICC stage
1	Adenocarcinoma	Rectum	IV
2	Adenocarcinoma	Splenic flexure	IV
3	Adenocarcinoma	Transverse colon	II A
4	Adenocarcinoma	Cecum	II A
5	Adenocarcinoma	Hepatic flexure	I
6	Adenocarcinoma	Ascending colon	I
7	Adenocarcinoma	Ascending colon	I
8	Adenocarcinoma	Sigmoid colon	II A
9	Mucinous adenocarcinoma	Rectum	IV
10	Adenocarcinoma	Descending colon	II A

UICC stages provided are based on pathological evaluation.

### 2.2 SRµCT imaging

X-ray phase-contrast tomography imaging was conducted using the SYRMEP beamline of the Italian synchrotron Elettra with the parallel white-beam configuration (mean spectrum energy 22 keV) and a sample-to-detector distance of 9 cm. The acquisition spanned 360°, with 3,600 projections and an offset centre of rotation to increase the field of view. For each projection, an exposure time of 20 ms was used, with a resulting pixel size of 2 µm. The paraffin blocks were mounted vertically and multiple acquisitions were performed with a 3.3-mm step height, resulting in a total scanning time of 3.6 min–6 min, depending on the number of vertical offset steps required. Each 3D dataset was reconstructed using SYRMEP Tomo Project (STP Version 1.6.3) with a single-distance phase retrieval algorithm (25), with a *δ-*to-*β* ratio of 50 and filtered back-projection. The vertical steps were stitched together using a custom Python 3 script. A detailed overview of SYRMEP capabilities and configurations is provided by Dullin et al. (26).

### 2.3 Histology

After SRµCT imaging, the FFPE tissue samples were sliced using a standard microtome (HM 340 E microtome, Thermo Fisher Scientific) into 2-µm-thick sections. Subsequently, they were deparaffinised at 60°C and rehydrated with an ascending ethanol series (xylene, isopropanol, 98%, 75%, 60% EtOH, dH_2_O; each 5 min). Histochemical staining was performed in accordance with the manufacturer’s instructions (HE; Merck). The slides were digitised with 5× tiled acquisition and stitched together (Axiovert 200, Zeiss).

### 2.4 Data visualisation

The 3D µCT datasets were rendered using VG Studio (V. 3.2.1). Identification of the corresponding histologic cutting plane, reslicing of the dataset, and registration were performed in accordance with the method described previously (27). Segmentation of relevant structures was performed manually with a drawing tablet (Wacom Cintiq 22) using the 3D “pen” tool in VG Studio. The pen allows the manual delineation of structures within grey value limits. Segmented structures were extracted and rendered individually with different colour render settings.

## 3 Results

In this study, 10 paraffin-embedded non-labelled human CRC samples (**Table 1**) were scanned using SRµCT in the whole paraffin block configuration, with a 2-µm voxel size and a horizontal offset to increase the effective field of view. All reconstructed SRµCT volumes displayed good contrast delineation between tissue interfaces, allowing for differentiation of the tissue architecture.

**Figure 1:**
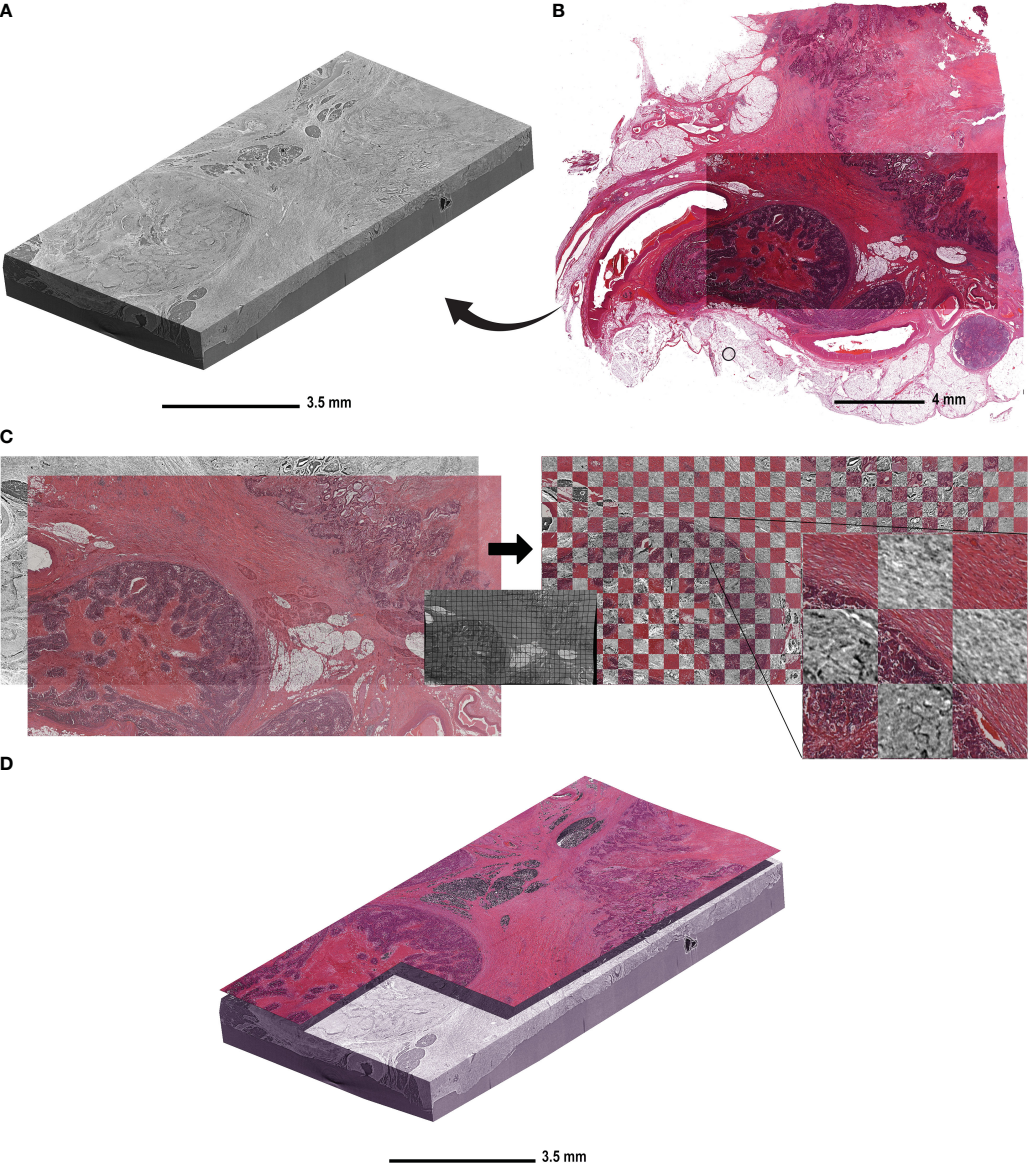
The workflow of histologic slide registration in the context of the 3D µCT dataset. **(A)** A representative reconstructed and rendered SRµCT volume of a descending colon adenocarcinoma sample (patient 10) embedded in paraffin. **(B)** The HE-stained slice acquired from the same block after the µCT imaging was complete. **(C)** The HE-stained section of the same CRC sample was registered to the virtual cutting plane in the µCT dataset. The virtual cutting plan was used as an undistorted ground truth and the HE-stained slice was non-rigidly deformed to compensate for the deformation due to cutting and histologic processing. The middle panel shows the same deformation matrix applied to a grid for better visualisation. The match post registration is shown on the right side with the checkerboard overlay, in which the continuity of the structures is seen between the HE-stained slice and the virtual cutting plane in CT. **(D)** The HE-stained slice is placed in the context of the µCT block in order to determine the “seed point” for structures.

**Figure 1** shows the reconstructed and stitched SRµCT acquisition of a specimen of descending colon adenocarcinoma tissue and the following registration with an HE-stained slice. The condition and time of storage of the paraffin blocks had no effect on image quality (**Figure 1A**). The HE-stained histologic sections were obtained after the SRµCT imaging session and showed the conserved and unaltered structure of both healthy and cancerous tissue. We did not observe any visible effects of the irradiation on the quality of the stained histologic images (**Figure 1B**). The virtual cutting plane in the SRµCT dataset corresponding to the HE-stained slice was approximated by manual alignment. Subsequently, the histologic image was warped using the corresponding SRµCT slice as the ground truth (**Figure 1C**). The precision of the registration is illustrated by the checkerboard overlay, in which continuity of major structures can be seen (**Figure 1C**, right). Finally, the registered histologic slice was placed in the 3D SRµCT dataset as a starting point to evaluate the structures in 3D.

**Figure 2:**
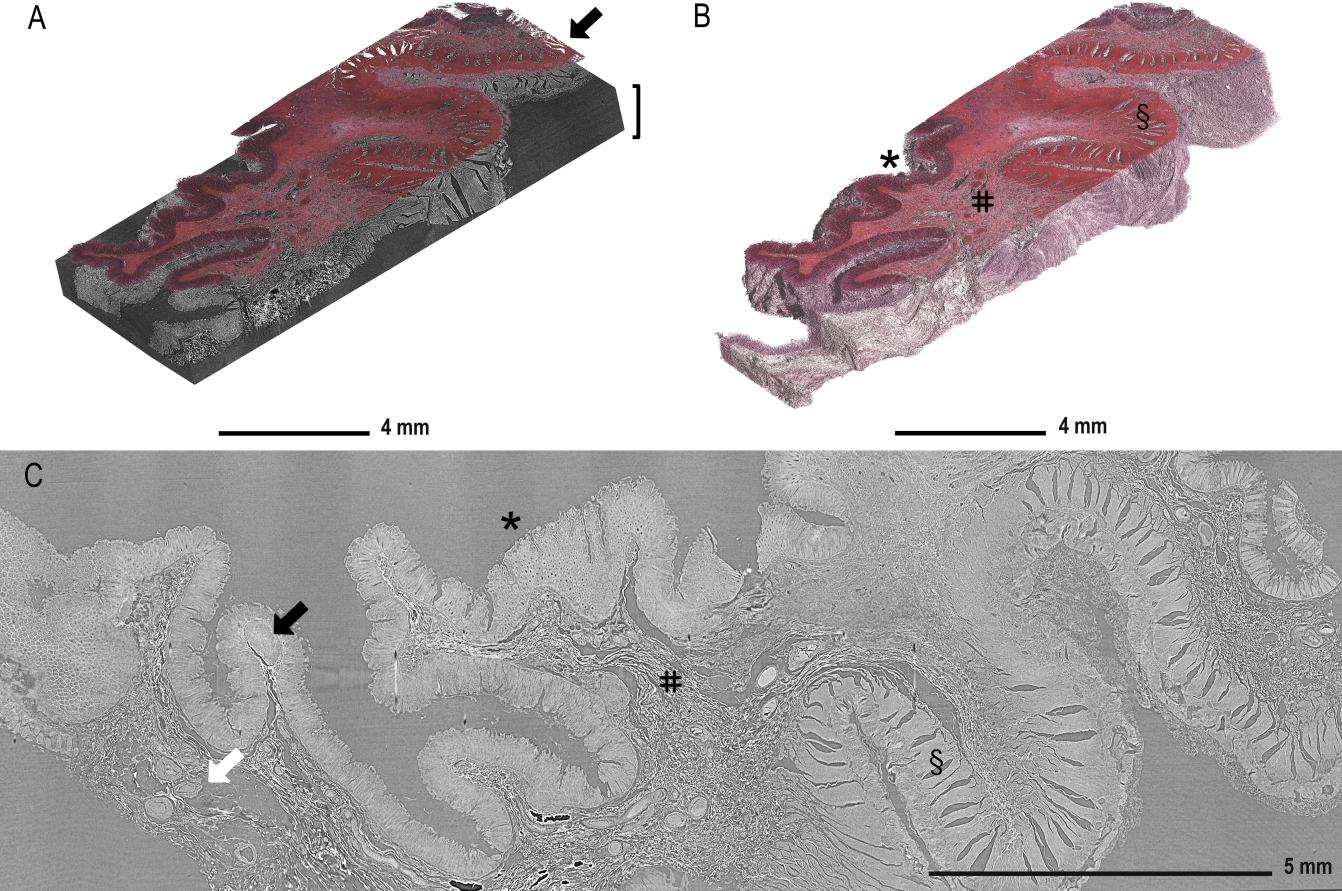
Demonstration of tissue features in registered µCT and HE-stained sections. **(A)** Overlay of the HE-stained slice (arrow) with virtual cutting position in the SRµCT dataset of an ascending colon adenocarcinoma sample (patient 7) in which the tissue is rendered together with the embedding material (brace). **(B)** The paraffin in the SRµCT dataset is rendered out exposing the 3D surface of the entire tissue piece. The tissue is artificially recoloured to mimic the HE palette. The collagen-rich submucosa is clearly delineated from the other tissues, as it is whiter in appearance. **(C)** All expected tissue types are recognised in the example 2D slice from the µCT dataset and in 3D (in B) with mucosa (*), submucosa (#), and muscularis (§) clearly visible and vessels (white arrow) and the lymph follicles (black arrow). Further examples of individual images from other FFPE blocks can be found in the **Supplementary Material**.

**Figure 2A** displays the reconstructed and stitched SRµCT volume of an ascending colon adenocarcinoma sample with a registered histologic HE-stained slice overlay (arrow) in the 3D context of the paraffin block (brace). **Figure 2B** displays a rendered SRµCT dataset in which paraffin was eliminated by thresholding. The CT dataset was then artificially recoloured to approximately match the overlaid HE histology. The grey value intensities can be represented by different artificial colours. Here, a pink-to-white gradient was used to denote low-to-high grey value transition. The colour delineation helped to discriminate the borders of regions rich in extracellular matrix, which generate higher contrast (white). Different tissue types, such as mucosa (*), submucosa (#), and muscularis (§), were easily identified in both 3D renderings (**Figure 2B**) and in individual slices (**Figure 2C**). Further examples of individual SRµCT slices from other CRC samples (**Figure S1**) and 3D renderings (**Figure S2**) can be found in the **Supplementary Material**.

**Figure 3:**
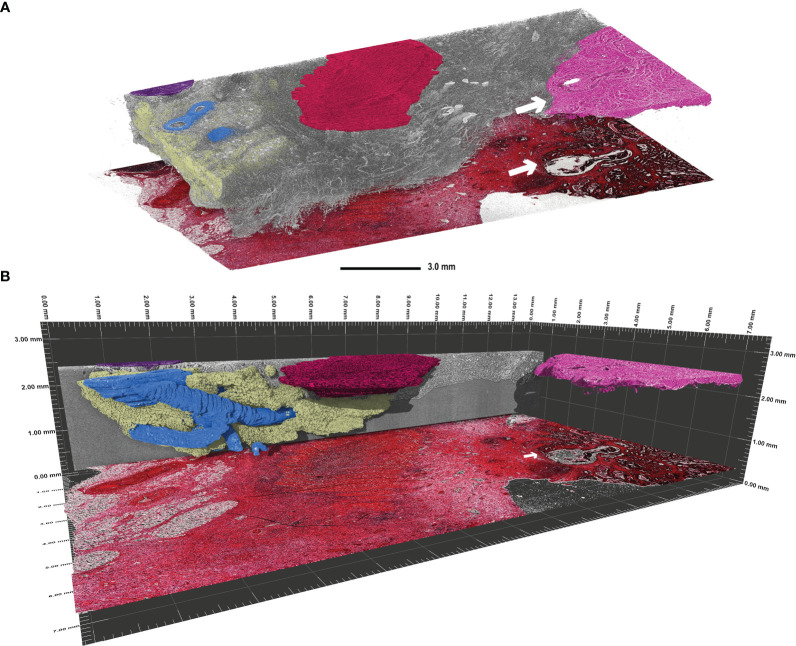
Visualisation of continuous 3D structures which were segmented using the histologic tissue slice as the “seed point” in a transverse adenocarcinoma sample (patient 3). **(A)** Segmentation of the main 3D structures inside the rest of the tissue (grey). Highlighted are the major vessel tree (blue), part of a lymph node (purple), fat tissue (yellow), muscle tissue (red), and the tumour tissue (pink). The tumour infiltration front is indicated by the white arrow in both the SRµCT dataset and the corresponding HE stained tissue slice. Evidently, this tumour has not penetrated the submucosal layer. **(B)** Side view of the segmented structures in perspective mode demonstrating the span of the 3D structures. Please note that the histologic slide was positioned with a vertical offset to facilitate depiction.

Furthermore, lymphatic follicles (**Figure 2C**, black arrow) and vessels (white arrow) can be identified and traced through the entire 3D volume, as demonstrated in **Figure 3**. 3D volume tracing was carried out using the histologic slide as the seed point for segmentation. Major structures such as fat tissue (yellow), a large vessel tree (blue), muscle tissue (red), a lymph node (purple), and tumour tissue (pink) can be identified in the volume. Segmentation of the tumour infiltration front provides a good representation of the extent to which tumour cells have invaded the surrounding tissue in the entire FFPE block, which would be challenging to assess in a single HE section. In this instance, the tumour evidently has not infiltrated the submucosa in any part of the volume (**Figure 3**).

## 4 Discussion

The primary aim of our study was to explore the feasibility of synchrotron radiation-based microcomputed tomography (SRµCT) for scanning human FFPE CRC samples obtained during surgical procedures in order to obtain a comprehensive 3D visual representation of the tissue’s microscopic architecture.

We demonstrate that SRµCT is an effective imaging tool for visualising CRC specimens in entire paraffin blocks. The combination of µCT and spatially registered histology enabled the assignment of specific staining information from the HE histology to the structural information in SRµCT. By applying the registration approach developed by Albers et al., we successfully placed a histologic slice into the 3D context of a SRµCT dataset (27). This combination offers a tangible advancement in visualising continuous 3D structures, such as the vessel tree and lymph nodes, while leveraging registered histologic slices as reference “seed points”. Furthermore, we show that the tumour tissue itself can be segmented within the tissue block, which enables assessment of the extent to which the tumour has invaded the surrounding tissue. The information obtained using SRµCT imaging may allow for a more comprehensive characterisation of CRC.

Accurate CRC staging is a crucial step for the prediction of patient outcomes and in the choice of appropriate therapy. In clinical practice, staging is performed based on a combination of immuno-/histochemistry of tumour biopsies and samples taken during surgical intervention. Despite its high level of sensitivity for features such as immune cells, collagen, and mucus, histology provides only a 2D representation of a single random slice in the resected tissue. Therefore, the heterogeneity of the paraffin-embedded specimens taken during surgical intervention is vastly undersampled. Expanding this representation can be achieved using other 3D imaging approaches. Serial cutting can be used to reconstruct a number of histologic slices into a 3D volume. However, this approach is not efficient and can be labour-intensive and time-consuming. Furthermore, the cutting process introduces non-rigid deformation in the cutting plane. This, in combination with non-uniform block cooling and multiple cutting attempts, means that the registration and reconstruction processes are computationally intensive (28, 29). In addition, the resulting 3D representation typically has non-isotropic resolution. An alternative method for 3D tissue visualisation is light sheet microscopy (LSM), which can offer 3D datasets with specific antibody stainings for structures of interest. LSM does require a designated sample preparation protocol, including tissue clearing and staining procedures if specific labelling is desired. For optimal results, different tissue types require optimised staining protocols in order to improve antibody diffusion (30, 31). Circumventing complex staining procedures while retaining some specific structural information can be achieved by third- and second-harmonic generation microscopy. This method generates specific signals for structures such as collagen and myosin without the need for labelling. Depending on the properties of the excitation laser, the maximum possible imaging depth can range between 100 µm and 1,000 µm; however, this method performs poorly in paraffin-embedded tissue (32). While the contrast produced in SRµCT is not specific to histologic features, the placement of the histologic slice into the 3D µCT context, as shown here, provides sufficient “starting point” information for extrapolation, which is a good alternative to the aforementioned methods.

By using a synchrotron facility featuring a coherent X-ray beam and high photon flux, we achieved rapid whole-block scanning times with enough soft-tissue contrast to effectively differentiate tissue interfaces. SRµCT of CRC has been explored previously by Takeda et al., who acquired a single tomogram of a metastatic colon carcinoma sample with a small field of view (5 × 5 mm^2^) and compared it to light microscopy images (33). In their µCT volume, they were able to recognise the necrotic area and areas of accumulated T cells; at the time, however, the maximum resolution reached was 30 µm, which is inferior to our small voxel size of 2 µm. Others have also explored whole-paraffin-block scanning, but with the use of conventional laboratory X-ray sources (34). Sakamoto and colleagues performed imaging of samples containing gastrointestinal lesions, obtained through endoscopic submucosal dissection. Whole-block FFPE tissue and fresh whole tissue were stained with Lugol’s iodine solution and imaged. Although imaging of fresh whole-tissue samples took 10–15 min, it did not display sufficient contrast (35). Pre-stained FFPE block imaging yielded good contrast but required 4–5 h per block to complete. In both cases, CT imaging has helped to assess whether R0 margins have been achieved in all specimens, but has failed to provide information additional to that already obtained by histology. However, similar to our results, FFPE whole-block imaging helped to assess the submucosal invasion of cancer in 3D. Yoshida et al. conducted a similar study imaging FFPE tissue obtained by rectal cancer resection, showing that whole-block imaging allows the visualisation of the circumferential resection margin and enables differentiation between metastatic and benign lymph nodes. Their imaging approach yielded better image quality than the aforementioned studies; however, each scan required approximately 8 h (36).

Although no polyps were observed in the random collection of samples examined in our study, we believe that polyps can be effectively examined using SRµCT due to the complete 3D overview of the tissue that the method provides. Since the paraffin embedding material can be successfully removed from the dataset, the topology of the mucosal surface in 3D can be investigated, which cannot be achieved via histology. Similar to the findings presented in previous reports, we were not able to show the presence of vascular invasion or tumour budding in the examined samples (33, 35, 36). At the utilised voxel size of 2 µm, this is a challenging task. Although the resolution can be increased, this usually decreases the effective field of view and means that a larger number of scans are required to cover the same area. In addition, due to the geometry of the paraffin block, if the field of view is significantly smaller than the longest block dimension, the non-scanned tissue and the plastic cassette will introduce artefacts into the µCT volume. As a result, many researchers choose to selectively extract tissue cylinders 1 mm–8 mm in size from the whole paraffin block to decrease the voxel size (1 µm–650 nm) at the expense of the field of view (37, 38). This naturally limits analysis of the tissue to the subsampled area extracted, which is not a suitable approach for large heterogeneous specimens such as CRC-resected tissue. In comparison, our FFPE whole-block scanning approach allows for simultaneous view of up to 8 × 8 × 3.5 *n* mm^3^, in which *n* is the number of consecutive offset scans at a pixel size of 2 µm. Some known facilities specialise in targeted zoom-in acquisition of fixed biological tissue ranging from whole organs to small biopsies, as well as high-throughput acquisition (26, 39, 40). However, this comes with the burden of data processing, storage, and visualisation, which may require specialised hardware. In addition, the large volumes of data generated can be problematic for an average user.

Histology and immunohistochemistry are considered to be the gold standard for diagnostic workup. The current state of the art in 3D imaging techniques, including SRµCT, cannot be considered clinically routine tools at this time and do not suffice to replace histology altogether. For the use of µCT diagnostics to be effective, several limitations have to be addressed. Although SRµCT offers excellent soft-tissue contrast and rapid scanning times, access to the facilities is restricted by beamline schedules and designated research proposals are required for beam use. Continuous efforts to translate phase-contrast advantages into smaller laboratory setups are being made (41–43). Further advancements in scanning times, usability, and standardisation are needed. Therefore, µCT imaging, as an immediate diagnostic tool, is limited by either the long scanning times offered by current state-of-the-art laboratory setups or the lack of accessibility to synchrotron facilities.

Nevertheless, SRµCT is a promising tool to provide complementary information to the clinical workup. SRµCT can be effectively applied to archived routinely obtained paraffin tissue blocks for research purposes. Since SRµCT is a non-destructive imaging technique, it can be easily integrated into multimodal imaging approaches. It therefore can be used as an anatomical reference guide or a means of targeted sampling in other downstream investigations. In addition, by imaging large numbers of archived FFPE blocks and adding the data to existing patient information, it is possible to improve classification and patient prognostic outcomes for future applications. A similar approach has been reported using archived HE-stained slices to predict patient survival by training a convolutional neural network (CNN) (44) to decompose complex tissue and provide prognostic staging. We hypothesise that adding the imaging features obtained by phase-contrast µCT whole-block scans to such clinical studies will significantly increase spatial FFPE block subsampling for the CNN, which cannot be performed using histology alone. This, in combination with existing histologic data, will greatly improve the overall performance of the CNNs. Furthermore, as evidenced in our study, a single histologic slice is enough for a pathologist to assign a label to a structure in the µCT dataset. Therefore, we speculate that histology/µCT single-slice pairs can be used to train a network for “style transfer” or virtual staining to artificially recolour entire volumes of µCT data. Similar approaches have been demonstrated recently for artificial staining of blank microscopy images of unstained tissue cuts. If such an approach was successful for µCT data, this could identify novel diagnostic imaging markers that may improve subclassification for optimal therapy and/or the accuracy of features for the prognostic outcomes of patients with CRC (45).

## 5 Conclusion

Phase-contrast µCT imaging is a powerful non-destructive imaging technique that combines high resolution, excellent contrast, and a high acquisition speed, and can be effectively used to image colon carcinoma resection tissue in great detail and provide information on many 3D features that would otherwise not be accessible by histology. This workflow can be effectively translated to other tissue types and archived samples to evaluate 3D features of various pathologies.

## Data availability statement

The raw data supporting the conclusions of this article will be made available by the authors, without undue reservation.

## Ethics statement

The studies involving humans were approved by the ‘Ethik-Kommission der Universitätsmedizin Göttingen’. The studies were conducted in accordance with the local legislation and institutional requirements. The participants provided their written informed consent to participate in this study.

## Author contributions

AS: Conceptualization, Data curation, Formal analysis, Investigation, Software, Validation, Visualization, Writing – original draft, Writing – review & editing. TG: Conceptualization, Data curation, Formal analysis, Investigation, Methodology, Validation, Writing – original draft, Writing – review & editing. JA: Formal analysis, Investigation, Visualization, Writing – review & editing. LD: Data curation, Investigation, Resources, Writing – review & editing. PN: Data curation, Investigation, Writing – review & editing. GT: Conceptualization, Resources, Writing – review & editing. HB: Conceptualization, Funding acquisition, Methodology, Project administration, Resources, Supervision, Writing – review & editing. FA: Conceptualization, Funding acquisition, Project administration, Resources, Supervision, Validation, Writing – review & editing. CD: Conceptualization, Data curation, Funding acquisition, Investigation, Methodology, Project administration, Resources, Supervision, Validation, Visualization, Writing – Original Draft, Writing – review & editing.

## Supplementary material

Supplementary Figure 1 Examples of individual 2D planes from the SRμCT datasets in an adenocarcinoma of the transverse colon from patient 3 **(A)**, of the splenic flexure from patient 2 **(B)**, of the cecum from patient 4 **(C)**, of the descending colon from patient 10 **(D)**.

Supplementary Figure 2 Example 3D renderings of an adenocarcinoma of the hepatic flexure from patient 5 **(A)** denoting mucosa (*), submucosa (#) and muscularis (§). Adenocarcinoma of the rectum from patient 1 **(B)** showing a major vessel (white arrow) and necrotic regions (black arrow). Mucinous adenocarcinoma of the rectum from patient 9 **(C)** with a segmented mucus pocket (pink) and cell agglomerates (purple).
