# Supplementary figures and images for: X-ray phase-contrast 3D virtual histology characterises complex tissue architecture in colorectal cancer
Source: Front Gastroenterol (Lausanne). 2023 Oct 24;2:1283052. doi: 10.3389/fgstr.2023.1283052 (PMC12952374; doi:10.3389/fgstr.2023.1283052)

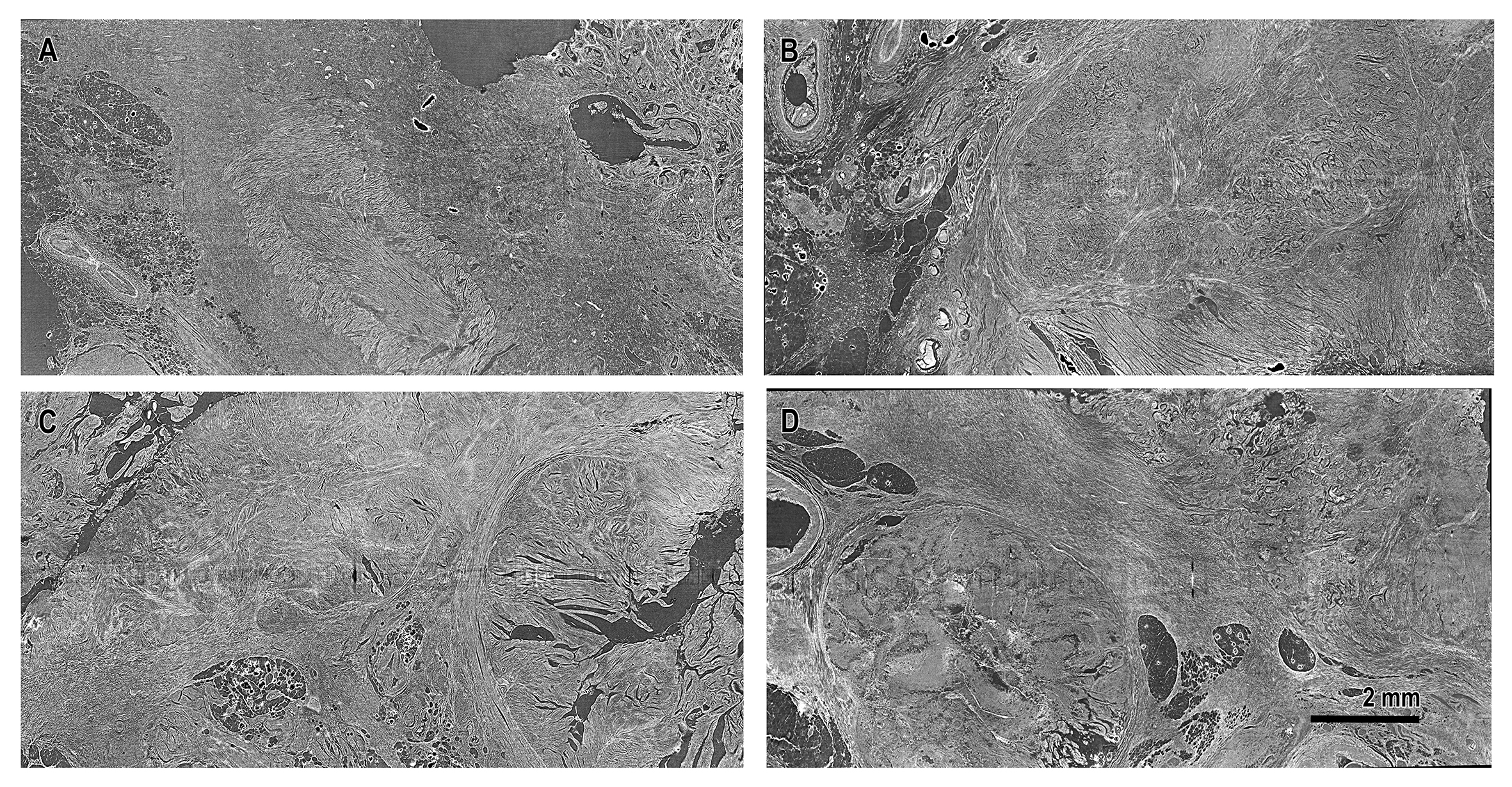

Supplement: Supplementary Figure 1 — Examples of individual 2D planes from the SRμCT datasets in an adenocarcinoma of the transverse colon from patient 3 (A), of the splenic flexure from patient 2 (B), of the cecum from patient 4 (C), of the descending colon from patient 10 (D). [file Image_1.jpeg]

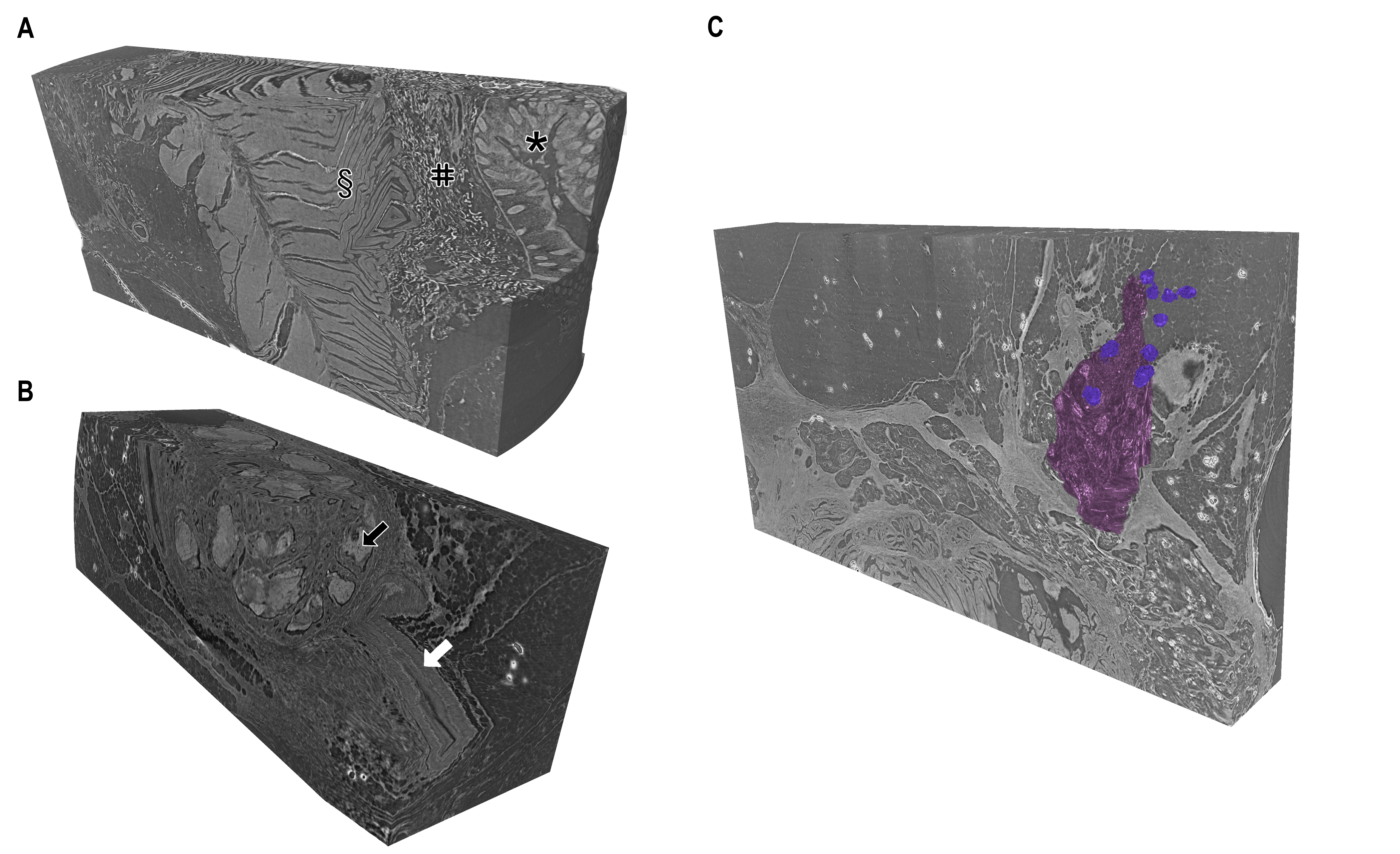

Supplement: Supplementary Figure 2 — Example 3D renderings of an adenocarcinoma of the hepatic flexure from patient 5 (A) denoting mucosa (*), submucosa (#) and muscularis (§). Adenocarcinoma of the rectum from patient 1 (B) showing a major vessel (white arrow) and necrotic regions (black arrow). Mucinous adenocarcinoma of the rectum from patient 9 (C) with a segmented mucus pocket (pink) and cell agglomerates (purple). [file Image_2.jpeg]
